# Assembly and Comparative Analysis of the Complete Mitochondrial Genome of *Corydalis ophiocarpa* (Papaveraceae)

**DOI:** 10.3390/cimb48060614

**Published:** 2026-06-12

**Authors:** Ming Lei, Cui Li, Jing Wang, Mei Qin, Li-Rong Huang, Xia-Lian Ou, Liang Kang, Han Liu, Zhan-Jiang Zhang

**Affiliations:** 1 Guangxi Key Laboratory of Medicinal Resources Protection and Genetic Improvement, Guangxi Botanical Garden of Medicinal Plants, Nanning 530023, China; leiming@gxyyzwy.com (M.L.); licuicui941@163.com (C.L.); 18565756016@139.com (J.W.); qinmei20210630@163.com (M.Q.); huanglirong2066@126.com (L.-R.H.); 18290045835@163.com (X.-L.O.); 17736623451@163.com (L.K.); 2 National Center for Traditional Chinese Medicine (TCM) Inheritance and Innovation, Guangxi Botanical Garden of Medicinal Plants, Nanning 530023, China; 3 Guangxi Engineering Research Center of TCM Resource Intelligent Creation, Guangxi Botanical Garden of Medicinal Plants, Nanning 530023, China; 4 School of Pharmacy, Guangxi Medical University, Nanning 530021, China; 5 Guangxi Key Laboratory for High-Quality Formation and Utilization of Dao-di Herbs, Guangxi Botanical Garden of Medicinal Plants, Nanning 530023, China

**Keywords:** *Corydalis ophiocarpa*, mitochondrial genome, multipartite architecture, RNA editing, mitochondrial plastid DNAs, phylogenetic analysis, benzylisoquinoline alkaloids

## Abstract

*Corydalis ophiocarpa* is a medicinally valuable plant, noted for its abundant alkaloid content. Despite its significance, the mitochondrial (mt) genome of this plant has not been characterized, which impedes both the phylogenetic understanding within the *Corydalis* genus and the comprehension of its full genetic potential. In this research, we successfully assembled the complete mitogenome of *C. ophiocarpa* by employing a hybrid method that integrates Oxford Nanopore long reads with Illumina short reads. The assembled genome forms a circular structure of 600,064 bp, with a GC content of 46.49%, and includes 63 genes, comprising 40 unique protein-coding genes (PCGs), 20 tRNAs, and three rRNAs. Through assembly and coverage analysis, we identified a 6383 bp forward repeat associated with a contig having approximately double the depth, indicating a repeat-mediated multipartite structure where the main circle may coexist with two smaller subgenomic forms. We discovered 775 C-to-U RNA editing sites across the 40 PCGs, with 95.4% being non-synonymous and favoring hydrophobic amino acid substitutions, particularly in Complex I subunits. Furthermore, we identified sixteen mt plastid DNA fragments constituting 2.43% of the mitogenome, a proportion more than double that found in the closely related *C. saxicola*. Phylogenetic analysis confirms that *C. ophiocarpa* is most closely related to *C. saxicola*, with *C. pauciovulata* as another close relative. This study presents the first complete mitogenome of *C. ophiocarpa*, providing a genomic basis for investigating the relationships between mt genome structure, post-transcriptional regulation, and specialized metabolism in the *Corydalis* genus.

## 1. Introduction

Mitochondria are semi-autonomous organelles characterized by their own genome and a specialized genetic system for replication, transcription, and translation, which are considered direct evidence of their endosymbiotic origin from an α-proteobacterial ancestor [1]. In stark contrast to their animal counterparts, land plant mitochondrial (mt) genomes are distinguished by remarkable complexity and variability [2]. This is demonstrated by their extensive size range [3], dynamic physical structures [4], and unique patterns of sequence evolution [5]. These genomes can vary in size by more than two orders of magnitude, from approximately 66 kb in the parasitic angiosperm *Viscum scurruloideum* to over 11 Mb in *Silene conica* [6]. This substantial physical expansion is not driven by gene acquisition; the core set of protein-coding genes (PCGs) remains largely stable and small (typically < 50), while the genomic space is predominantly occupied by non-coding DNA and sequences acquired from other cellular compartments, such as the chloroplast (cp) and nucleus [7,8]. Furthermore, plant mitochondrial (mt) genomes exhibit a distinctive evolutionary dynamic: their nucleotide sequences evolve at a very slow rate, yet they undergo frequent and rapid structural rearrangements [9]. Homologous recombination mediated by repetitive sequences is widely recognized as a central mechanism driving the structural dynamics of plant mt genomes. However, the precise architecture of these recombinogenic repeats, their genomic distribution, and their capacity to generate structural complexity remain to be fully characterized in many key lineages.

The genus *Corydalis* DC. comprises approximately 530 species [10], predominantly found in northern temperate and alpine regions, with its greatest diversity concentrated in the biodiversity hotspots of the Qinghai–Tibet Plateau and Hengduan Mountains [11]. Despite its significant horticultural and medicinal importance [10,12], the infrageneric phylogenetic relationships within *Corydalis* remain contentious due to extensive morphological plasticity [13], underscoring the need for molecular data to clarify these evolutionary relationships. To date, genomic studies on *Corydalis* have predominantly concentrated on the cp genome, leaving the evolutionary dynamics of its mt genome—such as structural rearrangements, gene content variation, and RNA editing patterns—largely unexplored [10,14,15]. This gap in genomic understanding is particularly significant for a genus renowned for its diverse array of benzylisoquinoline alkaloids [16], whose biosynthesis relies on precursor molecules from central carbon metabolism [17,18]. Mitochondria are central to this metabolic network, as the tricarboxylic acid cycle produces carbon skeletons that act as direct substrates for crucial steps in alkaloid pathways [17,18]. Consequently, the functional state of mitochondria, determined by their genome’s sequence, structure, and RNA editing patterns, may influence precursor availability, and thus affect alkaloid biosynthesis. Therefore, characterizing the mitogenome of *Corydalis* provides a critical opportunity to investigate how mitochondrial genomic variation is related to metabolic diversification in this lineage.

Within this genus, *Corydalis ophiocarpa* Hook. f. & Thomson is distinguished as a perennial medicinal herb of significant interest [19]. It is among the most extensively distributed species within the genus, spanning regions from East Asia to the Himalayas [19]. Notably, it possesses a well-documented history in Tibetan medicine, and its diverse benzylisoquinoline alkaloid profile (e.g., protopine, allocryptopine, berberine, and sanguinarine) has been pharmacologically characterized for its roles in promoting circulation and alleviating pain, among other activities [20,21]. These characteristics, coupled with the availability of mitogenomes from two closely related congeners (*C. saxicola* and *C. pauciovulata*), render *C. ophiocarpa* an ideal candidate for investigating the connections between mt genomic variation and medicinal alkaloid diversity.

In this study, we present the inaugural complete mt genome sequence of *C. ophiocarpa*, achieved through a hybrid assembly strategy that integrates Oxford Nanopore long reads with Illumina short reads. Our objectives were to: (1) characterize the fundamental features of the *C. ophiocarpa* mitogenome, including its gene content, repeat landscape, codon usage bias, and RNA editing sites; (2) identify mt plastid DNA (MTPT) fragments to assess inter-organellar sequence transfer events; and (3) determine its phylogenetic position within *Corydalis* and the broader Papaveraceae family through comparative genomic analyses. This work provides essential genomic data for future research on the conservation genetics and evolutionary biology of *C. ophiocarpa* and its relatives, and lays a foundation for exploring the potential link between mt genomic features and the diversity of benzylisoquinoline alkaloid biosynthesis.

## 2. Materials and Methods

### 2.1. Plant Materials, DNA and RNA Extraction and Sequencing

The plant material utilized in this study was authenticated as *C. ophiocarpa* by Drs. Zhan-Jiang Zhang and Cui Li of the Guangxi Botanical Garden of Medicinal Plants. High-molecular-weight genomic DNA was extracted from approximately 1 g of fresh leaf tissue, collected from greenhouse-grown plants, using the cetyltrimethylammonium bromide method [22]. This qualified DNA was employed to construct both paired-end Illumina and Oxford Nanopore sequencing libraries and served as the template for subsequent polymerase chain reaction (PCR) assays. The raw sequencing reads have been deposited in the NCBI Sequence Read Archive (SRA) under BioProject PRJNA1473575. The complete assemblies of the mt and cp genome have been submitted to GenBank, accessible under accession numbers PZ380598 for the mitogenome and PZ405204 for the plastome.

### 2.2. Assembly and Annotation of Mt Genomes

The mt genome was initially assembled from Nanopore long reads utilizing Flye (v2.9.1-b1780) with default parameters [23]. Putative mt contigs were identified through BLASTn searches using conserved *Arabidopsis thaliana* mt genes as queries [24]. To address the complex repetitive structures characteristic of plant mitogenomes, a hybrid scaffolding and polishing approach was employed. Illumina short reads were mapped to the candidate contigs using BWA (v0.7.17-r1118) to correct residual sequencing errors [25], while Nanopore long reads were aligned with minimap2 (v2.24-r1122) to manually verify and bridge repeat-associated branching points [26]. The final circular conformation and the alternative subgenomic structures were validated by visually inspecting the assembly graph in Bandage (v0.8.1) and confirming that all junctions were spanned by multiple spanning reads [27]. Junctions supported by fewer than three independent Nanopore reads were discarded, and no conflicting connections remained after this filtering.

For the annotation process, we employed GeSeq (v2.03) using the mt genome of Liriodendron tulipifera (NC_021152.1) as the reference, selected due to its extensive gene repertoire among angiosperms [28]. Predictions from the PMGA web server (http://www.1kmpg.cn/pmga/, accessed on 12 March 2023) were integrated to enhance and corroborate the GeSeq annotations. This integrative approach resulted in a consensus set of 40 PCGs. Any PCG present in the *L. tulipifera* reference but not initially identified was subjected to a targeted BLASTn search (v2.13.0; -evalue 1 × 10^−10^) against our assembly [24]. Transfer RNA genes were identified using tRNAscan-SE (v2.0.11) [29], and all gene models were manually reviewed and corrected for accuracy using Apollo (v1.11.8) [30].

### 2.3. Analysis of Codon Usage and Repeated Sequences

Protein-coding sequences were extracted from the mitogenome utilizing PhyloSuite v1.1.16 [31]. Codon usage bias was evaluated by calculating the relative synonymous codon usage (RSCU) values using MEGA7 [32]. Repetitive sequences were identified through the application of three specialized programs: MISA v2.1 [33] for simple sequence repeats (SSRs), Tandem Repeats Finder (TRF) v4.09 [34] for tandem repeats (TRs), and REPuter (https://www.cebitec.uni-bielefeld.de/bibiserv.cebitec.uni-bielefeld.de/, accessed on 14 March 2023) [35] for dispersed repeats. The results were compiled and visualized using Microsoft Excel 2021 for data organization and the Circos package v0.69-9 [36] for graphical representation.

### 2.4. Identification of MTPTs and RNA Editing Events

The cp genome of *C. ophiocarpa* was assembled with GetOrganelle v1.7.7.0 [37] and annotated using CPGAVAS2 (http://www.herbalgenomics.org/cpgavas2, accessed on 16 March 2023) [38], with annotations subsequently refined in CPGView (v2019.3.31) (http://www.1kmpg.cn/cpgview, accessed on 18 March 2023) [39]. To identify sequences potentially transferred between organelles, homologous regions between the mt and cp genomes were detected by BLASTn v2.13.0 [24] and visualized with Circos v0.69-9 [36]. Putative RNA editing sites were predicted with Deepred-mt (https://github.com/aedera/deepredmt, accessed on 19 March 2023) [40] using a cutoff of 0.9.

### 2.5. Phylogenetic and Synteny Analyses

Mt genome sequences of closely related species were retrieved from the NCBI database (accessed on 4 August 2024). Shared PCGs were extracted using PhyloSuite v1.1.16 [31]. For the purpose of phylogenetic reconstruction, nucleotide sequences of these genes were aligned with MAFFT v7.505 [41] and subsequently concatenated. A maximum-likelihood tree was inferred from the concatenated alignment employing IQ-TREE v1.6.12 [42] with 1000 bootstrap replicates. The phylogenetic tree was visualized using iTOL v6 [43].

To evaluate interspecific collinearity, we conducted BLASTn searches (https://ftp.ncbi.nlm.nih.gov/blast/executables/blast+/, accessed on 21 March 2023) [24] between the mt genome of *C. ophiocarpa* and those of related species, employing the following stringent parameters: -evalue 1 × 10^−5^, -word_size 9, -gapopen 5, -gapextend 2, -reward 2, -penalty 3. Only homologous regions exceeding 500 bp were retained as candidate collinear blocks for subsequent analysis. Multiple-genome synteny was visualized using MCScanX (b1ca533) [44].

### 2.6. Validation of Assembly Coverage and GC Content

To assess the sequencing depth and GC content of the final mitogenome assembly, raw reads were mapped to the reference sequence. For Illumina paired-end short reads, the reference genome was indexed, and reads were aligned using BWA (v0.7.17) with default parameters [25]. For Oxford Nanopore long reads, the reference genome was indexed, and reads were aligned using minimap2 (v2.24) with the map-ont preset [26]. The alignments were then converted to sorted BAM format and indexed using SAMtools (v1.9) [45]. The sorted BAM files for both datasets served as input for Qualimap v2.2.2-dev, which generated sliding-window (1-kb window, 500-bp step) coverage depth, and GC content profiles across the reference genome [46]. These resulting coverage plots were visualized as linear graphs across the entire mitogenome.

## 3. Results

### 3.1. General Features of C. ophiocarpa Mt Genome

**Figure 1:**
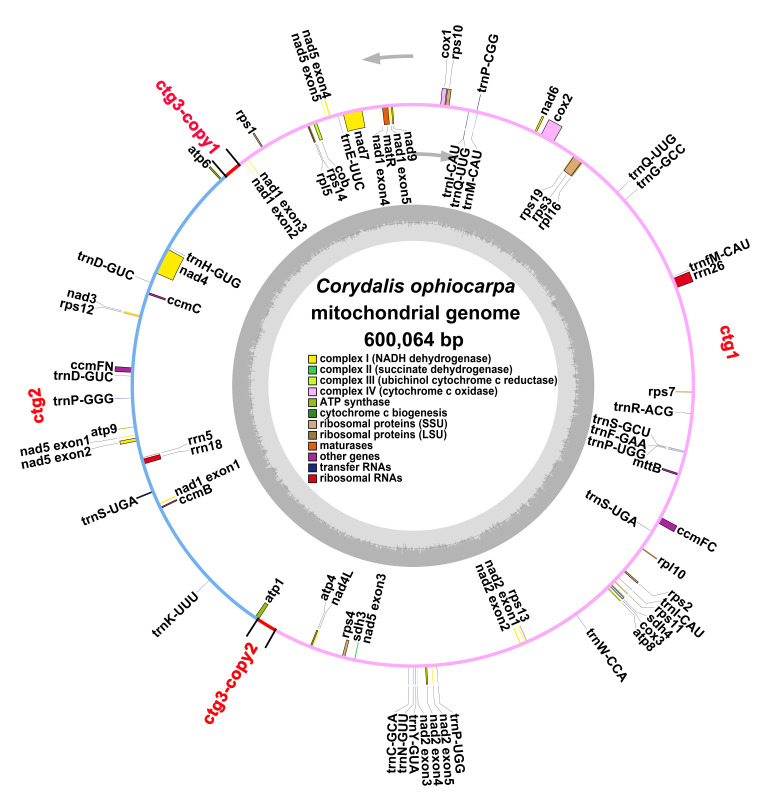
A schematic representation of the *C. ophiocarpa* mitochondrial (mt) genome. The inner circle displays the GC content distribution, with dark grey shading representing GC content and a light black line indicating the 50% GC threshold. The outer circle illustrates the circularized genomic sequence, with thick lines in pink, blue, and red signifying the assembled contigs ctg1, ctg2, and ctg3, respectively. Black vertical lines demarcate the boundaries of each contig. It is noteworthy that the red block (ctg3) appears twice, signifying a duplicated repeat region within the genome. Genes positioned on the interior and exterior of the outer circle are transcribed in a clockwise and counterclockwise direction, respectively.

We sequenced and assembled the complete mt genome of *C. ophiocarpa* utilizing a hybrid approach that integrates Oxford Nanopore long reads and Illumina short reads. The initial assembly yielded an assembly graph consisting of three contigs (ctg1: 413,651 bp; ctg2: 173,647 bp; ctg3: 6383 bp), indicative of the structural complexity arising from extensive repetitive sequences. To resolve this graph, we conducted long-read scaffolding by aligning the flanking sequences of branching contigs to Nanopore reads with minimap2 (v2.24) [26]. A junction was considered validated only when it was continuously spanned by a minimum of three independent reads. This methodology resolved the assembly into a single, circular chromosome of 600,064 bp with a GC content of 46.49% (Figure 1; GenBank accession: PZ380598). This length is consistent with the retention of two copies of the 6383 bp repeat in the master circle, a conclusion corroborated by the elevated coverage observed for ctg3.

To assess the assembly coverage and GC content distribution, both Illumina short reads and Oxford Nanopore long reads were aligned to the final mitogenome. The Illumina reads exhibited an average coverage depth of 693×, while the Oxford Nanopore reads demonstrated an average coverage depth of 237×. The average GC contents were 45.86% for Illumina and 46.53% for the Oxford Nanopore reads (Figure S1).

Coverage analysis revealed a significant disparity among contigs: ctg3 exhibited a mean depth of 398×, approximately 1.9-fold and 1.8-fold higher than that of ctg1 (208×) and ctg2 (224×), respectively (Figure S2, Table S1). This nearly twofold increase in coverage suggests the presence of repetitive sequences, indicating that ctg3 represents such a region. In plant mitochondria, these repeats are recognized as hotspots for homologous recombination [4]. Based on this observation, we hypothesize that this repeat facilitates dynamic conformational shifts. Consequently, alongside the master circle, the genome may also exist in an alternative conformation comprising two smaller, separate circular molecules (Figure S2). This structural versatility could underlie the observed genomic plasticity of the *C. ophiocarpa* mitogenome. The annotation of the assembled genome identified 63 genes, including 40 unique PCGs, 20 transfer RNA (tRNA) genes, and 3 ribosomal RNA (rRNA) genes (Table 1). Among the PCGs, 24 were core mt genes, while 16 were variable. All PCGs and rRNA genes, along with most tRNA genes, were present as single copies. In contrast, five tRNA genes (*trnD-GUC*, *trnI-CAU*, *trnP-UGG*, *trnQ-UUG*, and *trnS-UGA*) were found in two copies (Table 1). Among the 63 annotated genes, nine contained introns, resulting in multiple exons. Specifically, three genes (*ccmFC*, *rps3*, and *rps10*) contained two exons, cox2 contained three, nad4 contained four, and four genes (*nad1*, *nad2*, *nad5*, and *nad7*) contained five exons (Table S2).

In order to identify potential MTPT transfers, we sequenced and assembled the complete cp genome of *C. ophiocarpa*. The assembly yielded a circular molecule of 200,540 bp, exhibiting the conserved quadripartite structure of typical plastid genomes: a large single-copy (LSC) region of 96,266 bp, a small single-copy (SSC) region of 8890 bp, and two inverted repeat (IR) regions of 47,692 bp each (Figure S3). Annotation revealed a total of 110 unique genes, comprising 77 PCGs, 29 tRNA genes, and 4 rRNA genes (Table S3). This complete cp genome sequence served as the essential reference for the subsequent comparative analysis aimed at identifying MTPT fragments within the *C. ophiocarpa* mitogenome.

### 3.2. Codon Usage of PCGs

To evaluate codon usage bias within the mt genome of *C. ophiocarpa*, we computed the RSCU values for its 40 distinct PCGs (Figure S4, Table S4). Methionine (AUG) and tryptophan (UGG), each encoded by a single codon, exhibited an RSCU of 1.0, while the remaining PCGs demonstrated significant codon usage bias. The extent of this bias varied considerably among amino acids. Notable preferences were identified for several amino acids; for instance, alanine was predominantly encoded by GCU (RSCU = 1.60), and glutamine exhibited a strong bias toward CAA (RSCU = 1.52). Conversely, phenylalanine showed only a slight codon preference, with its highest RSCU value below 1.2. Overall, this range of codon usage patterns suggests the combined influence of mutational bias and translational selection in shaping the evolution of the *C. ophiocarpa* mitogenome.

### 3.3. Repeated Sequences

SSR analysis revealed the presence of 151 repeats within the *C. ophiocarpa* mitogenome (Figure S5A; Table S5). These repeats included 36 mononucleotide, 31 dinucleotide, 17 trinucleotide, 62 tetranucleotide, 4 pentanucleotide, and 1 hexanucleotide SSR, with tetranucleotide repeats being the most prevalent, accounting for 41.06% of the total. Among the mononucleotide SSRs, adenine (A) homopolymers were predominant, constituting 40.00%, while TA/AT repeats were the most common among dinucleotide SSRs, representing 50.00%.

Extended repetitive structures were also characterized. Twenty-one TRs, ranging from 12 to 36 bp and with a sequence identity exceeding 76%, were identified (Figure S5B; Table S6). The analysis of dispersed repeats, with a minimum length of 30 bp, revealed 1002 repeat pairs, categorized as 494 palindromic, 506 forward, and 2 reverse repeats, with no complementary repeats detected (Figure S5B; Table S7). Notably, the longest forward repeat, measuring 6383 bp, corresponded precisely to the size of the major repetitive contig (ctg3), thereby providing direct sequence-based evidence that this region serves as a primary recombination site, which underlies the structural plasticity proposed in our model.

### 3.4. Identification of MTPTs and Synteny Analyses

**Figure 2:**
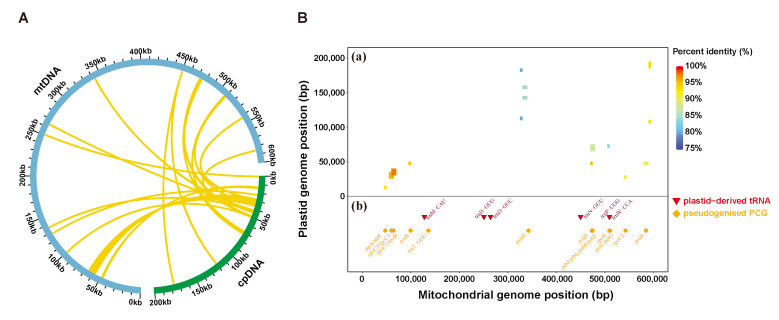
Analysis of homologous fragments in the chloroplast (cp) and mt genomes of *C. ophiocarpa*. (**A**) The schematic representation of homologous fragments in the cp and mt genomes of *C. ophiocarpa*. The blue arcs denote the mt genome, while the green arcs represent the cp genome. The yellow lines indicate homologous genomic fragments. (**B**) Analysis of DNA transfer from plastid to mitochondrion in *C. ophiocarpa*. (**a**) The heatmap illustrates the percent identity between the cp and mt genomes of *C. ophiocarpa*. Each colored block denotes a homologous fragment between the cp and mt genomes. The X-axis represents the position within the mt genome, while the Y-axis corresponds to the position within the cp genome. The color gradient, ranging from blue to red, signifies percent identity, with blue indicating approximately 75%, yellow approximately 85%, and red 100%. (**b**) Red triangles indicate the locations of tRNA genes transferred from the cp genome, whereas orange squares denote the positions of pseudogenized cp genomic counterparts.

A comparative analysis of the mt and cp genomes of *C. ophiocarpa* identified 16 plastid-derived DNA fragments integrated into the mitogenome. These MTPTs collectively span 14,589 bp, constituting 2.43% of the mitogenome, with six fragments exceeding 1 kb in length (Figure 2A,B; Table S8). Further annotation revealed that these fragments originated from various regions of the plastid genome, including PCGs, tRNAs, and intergenic spacers (Figure 2A,B; Table S8).

**Figure 3:**
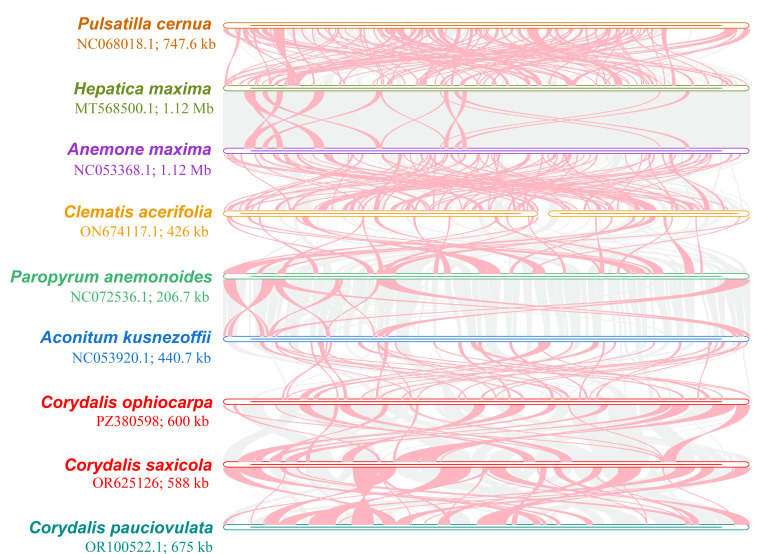
The synteny analysis of *C. ophiocarpa* and related mt genomes. The genomes are depicted as horizontal bars, with ribbons indicating homologous regions. Gray and red shading indicate areas of high conservation and inversions, respectively. Blocks smaller than 0.5 kb were excluded from the analysis. Regions that are not connected are specific to individual species.

To elucidate the structural evolution of the mitogenome, we conducted a comparative synteny analysis between the *C. ophiocarpa* mitogenome and those of eight related species. This analysis revealed significant syntenic conservation, with several collinear blocks shared among the genomes. The highest degree of synteny was observed between *C. ophiocarpa* and *C. saxicola*, followed by *C. pauciovulata*, highlighting their close phylogenetic relationships and structural stability (Figure 3). In contrast, several genomic regions were unique to *C. ophiocarpa*, exhibiting no synteny with the other species, suggesting a potential role in species-specific adaptations. Furthermore, the order and orientation of homologous blocks varied across the species, indicating extensive mt genomic rearrangements—such as inversions and translocations—that reflect their divergent evolutionary trajectories.

### 3.5. Phylogenetic Analyses

**Figure 4:**
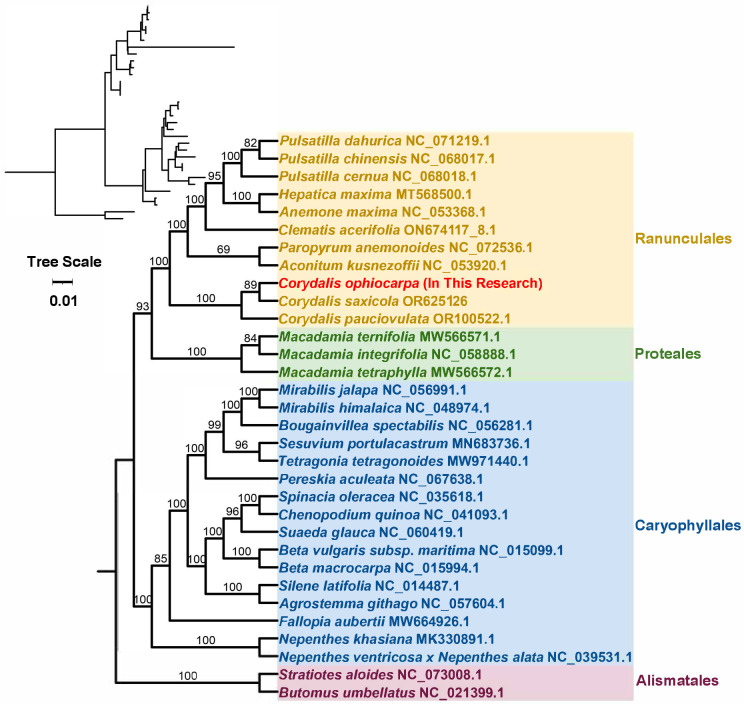
Phylogenetic analysis of *C. ophiocarpa* alongside 31 other species utilizing the nucleotide sequences of 23 conserved core mt PCGs. *S. aloides* and *B. umbellatus* were designated as outgroups. The numerical values at each node indicate the bootstrap probability.

To ascertain the phylogenetic placement of *C. ophiocarpa*, we conducted a phylogenetic analysis utilizing nucleotide sequences from 23 conserved mt PCGs. The dataset comprised 32 species from the orders Ranunculales, Proteales, and Caryophyllales, with two species from Alismatales (*Stratiotes aloides* and *Butomus umbellatus*) serving as outgroups (Table S9). The resultant maximum-likelihood tree (Figure 4) aligned with the current Angiosperm Phylogeny Group (APG) classification. Within *Ranunculales*, *C. ophiocarpa* was identified as the sister species to *C. saxicola*, and together, they constituted a clade sister to *C. pauciovulata*. This close phylogenetic relationship is corroborated by the high degree of mt synteny observed between *C. ophiocarpa* and *C. saxicola* (Figure 3).

### 3.6. RNA Editing Events in C. ophiocarpa

Using Deepred-mt (cutoff = 0.9), we predicted 775 C-to-U RNA editing sites across the 40 PCGs (Table S10). Of these sites, 739 (95.4%) were non-synonymous. The *nad4* gene exhibited the highest editing density with 67 sites, followed by *nad7* with 43 sites (Figure S6, Table S11). Additionally, premature termination codons were predicted in five genes: *atp6*, *ccmC*, *ccmFC*, *rps10*, and *rps11*. The total number of predicted editing sites in *C. ophiocarpa* (775) closely resembles that reported for the related species *C. saxicola* (779) [10].

## 4. Discussion

### 4.1. Structural Fluidity

Mitochondria in eukaryotes, hypothesized to have originated from aerobic bacteria engulfed by ancestral archaea [47,48], exhibit a double-membrane structure containing mt DNA, ribosomes, and RNA within nucleoids. Throughout evolution, most mt genes were either lost or transferred to nuclear genomes, resulting in significant variability in size and structure across species. Notably, plant mt genomes are considerably larger and exhibit more complex structural dynamics compared to those of animals [49]. Plant mt genomes range from approximately 66 kb to 12 Mb, whereas mammalian mt genomes typically measure 15–17 kb in length [50]. Furthermore, certain plant mt genomes can exist in linear, multi-branched and polycyclic structures [4,51,52,53]. In this study, the mt genome of *C. ophiocarpa* exemplifies the structural complexity and recombinational activity characteristic of land plant mitogenomes [7]. Notably, a 6383 bp forward repeat, the longest identified within this mitogenome, corresponds precisely to ctg3, which exhibits approximately twice the coverage compared to other contigs (Figure S2, Table S1). This congruence strongly supports the presence of a repeat-mediated multipartite architecture (Figure S2). A similar pattern of coverage disparity and tri-contig structure has been observed in *C. saxicola*, another species within the genus *Corydalis* [10], suggesting that repeat-driven structural dynamics are a conserved genomic feature within this lineage. This observation is consistent with the emerging consensus that plant mitogenomes are not static circular molecules but rather dynamic ensembles of alternative conformations generated by recombination among large repetitive elements [54]. Multipartite organizations have been experimentally confirmed in *Arabidopsis*, *Cucumis*, and *Brassica*, suggesting that the capacity to exist in multiple coexisting genomic conformations is phylogenetically widespread [4,54,55]. The functional importance of this recombinational potential, however, remains a subject of ongoing investigation. The hypotheses proposed in the literature include the modulation of respiratory gene copy number without alteration of the master circle [56,57], facilitation of DNA repair via homologous recombination [58], and a non-adaptive byproduct of frequent mt fusion and fission [59]. The current data do not allow for a definitive conclusion regarding which of these possibilities holds true, but one clear prediction emerges: if multipartite architecture serves an adaptive purpose, the abundance of subgenomic circles relative to the master genome should vary with tissue type, developmental stage, or environmental context. Such shifts could be monitored experimentally by quantitative real-time PCR (qRT-PCR) across the predicted recombination junction.

### 4.2. Inter-Organellar Transfer, Phylogenetic Placement, and Syntenic Conservation

The *C. ophiocarpa* mitogenome has incorporated a significant quantity of plastid-derived DNA (Figure 2A,B, Table S8). We identified 16 MTPT fragments spanning 14,589 bp, collectively constituting 2.43% of the mt genome (Figure 2A,B, Table S8). This proportion exceeds by more than double the 1.04% reported for the closely related species *C. saxicola* [10]. What accounts for this marked disparity between two congeneric species? The net accumulation of plastid DNA in the mt genome reflects the interplay of several processes, including the frequency of inter-organellar DNA transfer, the efficiency of sequence integration, and the duration of retention before deletion—each of which may vary among lineages [60]. Each of these parameters may vary independently, and even modest evolutionary divergence can produce measurable differences in MTPT content. Several non-mutually exclusive mechanisms may explain this elevated MTPT fraction in *C. ophiocarpa*. First, this species harbors a considerably more extensive repeat landscape than *C. saxicola*, including the 6383 bp recombinogenic repeat and 1002 dispersed repeat pairs. An abundance of repetitive elements can render the mitogenome more susceptible to recombination, which may in turn facilitate the insertion and long-term maintenance of plastid-derived sequences, potentially through DNA repair pathways such as microhomology-mediated end joining or double-strand break repair [4,58]. Second, differential rates of MTPT loss may contribute to the observed difference. Plant mitochondria are known to eliminate plastid sequences rapidly [60,61], and *C. saxicola* may simply excise such fragments more efficiently or more frequently. Additional factors, including the physical proximity of plastids and mitochondria within the cell and lineage-specific variation in organellar DNA repair efficiency, could further influence the net transfer rates. Distinguishing among these possibilities will require a broader sampling of mitogenomes across the genus *Corydalis*. Such data would enable a reliable estimation of lineage-specific insertion and loss dynamics, thereby clarifying the evolutionary forces that shape MTPT accumulation in this group.

The functional outcomes of integrated plastid sequences in *C. ophiocarpa* exhibit heterogeneity. Several MTPTs contain intact plastid tRNA genes (*trnD-GUC*, *trnH-GUG*, *trnN-GUU*, *trnP-UGG*, and *trnW-CCA*) that do not display obvious disruptive mutations (Figure 2B, Table S8). The absence of such mutations suggests that these tRNA genes may retain the ability to fold into functional cloverleaf structures. In *A. thaliana*, the plastid-derived *trnW-CCA* is essential for mt translation [62], illustrating how transferred tRNAs can be functionally integrated into the mt translational apparatus. Whether the homologous tRNAs in *C. ophiocarpa* are expressed and functional remains to be determined; however, their retention without apparent sequence decay suggests that they may be selectively maintained. In contrast, all transferred protein-coding sequences are partial pseudogenes (Table S8), a pattern fully consistent with the gradual sequence erosion that typically follows integration unless a functional role is acquired [10,11]. This dichotomy between the retention of tRNA genes and the decay of protein-coding sequences is a recurring pattern in plant mitogenome evolution and likely reflects the relative ease with which small, structurally autonomous tRNA genes can be integrated into the mt genetic system.

Beyond inter-organellar transfer, the phylogenetic positioning and syntenic organization of the *C. ophiocarpa* mitogenome offer further insights into its evolutionary trajectory. Phylogenetic reconstruction utilizing 23 conserved mt PCGs positions *C. ophiocarpa* as sister to *C. saxicola*, with *C. pauciovulata* forming a closely related clade (Figure 4). This topology aligns with previous cp-based phylogenies of the genus [10,11,61], and the concordance between the two organellar phylogenies underscores the close evolutionary relationship among these species, suggesting the absence of significant phylogenetic conflict due to differential organellar inheritance or introgression. Comparative synteny analysis further corroborates this relationship. Extensive collinear blocks are shared between *C. ophiocarpa* and *C. saxicola*, indicating that large-scale gene order has remained largely intact since the divergence of these two species (Figure 3). A high degree of synteny is also observed with *C. pauciovulata*, reflecting their close evolutionary relationship (Figure 3). Comparisons with more distantly related taxa, however, reveal accumulated inversions, translocations, and species-specific segments—a pattern consistent with the punctuated model of plant mitogenome evolution, characterized by extended periods of structural stasis interrupted by bursts of recombination-driven remodeling [4,57]. The relatively conserved synteny between *C. ophiocarpa* and *C. saxicola*, as well as with *C. pauciovulata*, suggests that these species diverged recently enough that large-scale rearrangements have not yet obscured their shared ancestral gene order. Nevertheless, the expanded repeat repertoire in *C. ophiocarpa* may foreshadow accelerated structural divergence over longer evolutionary timescales.

### 4.3. Implications for Alkaloid Metabolism and Future Directions

*C. ophiocarpa* synthesizes a diverse range of benzylisoquinoline alkaloids, a biosynthetic process that is dependent on mt ATP production and carbon skeletons derived from the tricarboxylic acid cycle [17,18]. Nonetheless, this proposed link remains speculative in the absence of direct functional evidence, and our current data merely provide a basis for formulating such hypotheses.

The predicted RNA editing counts in *C. ophiocarpa* (775 sites) and *C. saxicola* (779 sites) were nearly identical [10] (Figure S6, Table S10). Given the 65% validation rate reported for *C. saxicola* [10], the actual numbers are likely comparable, approximately 500 sites. This similarity suggests that the core RNA editing machinery is largely conserved between the two species. Several studies have demonstrated that RNA editing occurred in mt transcripts as a post-transcriptional modification mechanism, playing crucial roles in plant development, male sterile modulation, cell signaling regulation, and abiotic stress response [63,64,65,66,67,68,69]. RNA editing results in a majority of non-synonymous mutations and a few synonymous mutations, with the latter often considered biologically silent in plants. Interestingly, a recent study demonstrated that a synonymous mutation in an aminocyclopropane-1-carboxylic acid synthase gene contributes to cucumber fruit length domestication by regulating RNA structural conformations [70]. This finding highlights the hidden biological function of RNA editing as initiators of synonymous and nonsynonymous mutations. Whether these RNA editing events in mt genomes could lead to divergent alkaloid profiles among different species remains an open question. This hypothesis could be tested through parallel transcriptomic and metabolomic analyses.

Beyond RNA editing, the mitogenome of *C. ophiocarpa* exhibits several structural characteristics that may also have functional implications. The 6383 bp recombinogenic repeat, along with the 1002 dispersed repeat pairs and the tri-contig assembly graph, indicates a dynamic multipartite architecture (Table S7). Although the functional significance of this structural fluidity remains undetermined, it is possible that the relative abundance of subgenomic conformations could influence the copy number of respiratory genes located on those circles, thereby affecting the mt energy output. Furthermore, the significantly higher MTPT content in *C. ophiocarpa* (2.43%) compared to *C. saxicola* (1.04%) suggests lineage-specific differences in inter-organellar DNA transfer. Whether these structural and compositional variations correlate with metabolic traits—such as the capacity for alkaloid biosynthesis—remains an open question that necessitates the integration of mitogenomic, transcriptomic, and metabolomic data across multiple tissue types or growth conditions. As an initial step toward addressing this question, qRT-PCR across the predicted recombination junction in tissues with contrasting alkaloid accumulation could reveal whether multipartite conformation ratios vary in a manner consistent with a functional role.

Several important considerations must be acknowledged. The RNA editing sites identified in this study are based solely on computational predictions; experimental validation through qRT-PCR and Sanger sequencing is essential to confirm their authenticity and editing efficiency. Additionally, establishing a causal link between mitogenome architecture and alkaloid production necessitates genetic manipulation, which remains technically challenging in plant mitochondria despite recent advancements in organellar genome editing [71,72]. Furthermore, a broader sampling of mitogenomes across the genus *Corydalis* is required to ascertain whether the observed patterns are representative of the entire lineage.

Despite these limitations, the comparative framework delineated herein demonstrates how a fundamental characterization of mitogenome architecture and post-transcriptional modification can elucidate functional inquiries concerning lineage-specific metabolic diversity. The complete mitogenome of *C. ophiocarpa*, along with the comparative analyses presented in this study, establishes a foundation for future functional and evolutionary investigations of this medicinally significant genus.

## 5. Conclusions

This study presents the first complete mt genome of *C*. *ophiocarpa*. A recombinogenic repeat of 6383 bp, exhibiting twofold elevated coverage, reveals a dynamic multipartite genome architecture shared with *C. saxicola*. This suggests that repeat-driven structural fluidity may be conserved within the genus. The predicted RNA editing landscape is predominantly characterized by non-synonymous, hydrophobic-biased substitutions, accounting for 95.4% of 775 sites, with the highest density in Complex I subunits. Editing sites at the third codon position are exclusively synonymous, underscoring the functional specificity of the editing machinery. Notably, the editing repertoires of *C. ophiocarpa* and *C. saxicola* are nearly identical, indicating substantial conservation of the core post-transcriptional machinery. This conservation implies that any divergence in alkaloid profiles is more likely to result from nuclear-encoded metabolic genes. However, these predictions require experimental validation in future studies. Sixteen MTPT fragments highlight lineage-specific variation in inter-organellar DNA transfer. Phylogenetic analysis positions *C. ophiocarpa* as sister to *C. saxicola*, with *C. pauciovulata* as a close relative. Collectively, this study provides a foundational genomic resource for understanding mitogenome architecture, RNA editing, and specialized metabolism in the genus *Corydalis*.

## Figures and Tables

**Table 1:** A list of genes identified in the *C. ophiocarpa* mt genome.

	Gene Groups	Gene Names
Core genes	ATP synthase	*atp1*, *atp4*, *atp6*, *atp8*, *atp9*
	NADH dehydrogenase	*nad1*, *nad2*, *nad3*, *nad4*, *nad4L*, *nad5*, *nad6*, *nad7*, *nad9*
	Cytochrome *b*	*cob*
	Cytochrome *c* biogenesis	*ccmB*, *ccmC*, *ccmFC*, *ccmFN*
	Cytochrome *c* oxidase	*cox1*, *cox2*, *cox3*
	Maturases	*matR*
	Protein transport subunit	*mttB*
Variable gene	Ribosomal protein large subunit	*rpl5*, *rpl10*, *rpl16*
	Ribosomal protein small subunit	*rps1*, *rps2*, *rps3*, *rps4*, *rps7*, *rps10*, *rps11*, *rps12*, *rps13*, *rps14*, *rps19*
	Succinate dehydrogenase	*sdh3*, *sdh4*
rRNA genes	Ribosome RNA	*rrn5*, *rrn18*, *rrn26*
tRNA genes	Transfer RNA	*trnC-GCA*, *trnD-GUC* (×2), *trnE-UUC*, *trnF-GAA*, *trnfM-CAU*, *trnG-GCC*, *trnH-GUG*, *trnI-CAU* (×2), *trnK-UUU*, *trnM-CAU*, *trnN-GUU*, *trnP-CGG*, *trnP-GGG*, *trnP-UGG* (×2), *trnQ-UUG* (×2), *trnR-ACG*, *trnS-GCU, trnS-UGA* (×2), *trnW-CCA*, *trnY-GUA*

(×2) refer to genes with two copies.

## Data Availability

The raw Oxford Nanopore and Illumina sequencing reads have been deposited in the NCBI Sequence Read Archive (SRA) under BioProject PRJNA1473575. The complete mitochondrial and chloroplast genome sequences have been deposited in GenBank under accession numbers PZ380598 (mitogenome) and PZ405204 (plastome). These sequences are currently being processed and will be publicly released on 15 July 2026. All other data are contained within the manuscript and supplementary files.

## Supplementary Materials

The following supporting information can be downloaded at: https://www.mdpi.com/article/10.3390/cimb48060614/s1.
