# Supplementary material for: Assembly and Comparative Analysis of the Complete Mitochondrial Genome of Corydalis ophiocarpa (Papaveraceae)
Source: Curr Issues Mol Biol. 2026 Jun 12;48(6):614. doi: 10.3390/cimb48060614 (PMC13297740; doi:10.3390/cimb48060614)
Supplement: Supplementary file 1 [file cimb-48-00614-s001.zip › Supplementary Figures.pdf]

Assembly and Comparative Analysis of the Complete Mitochondrial Genome of *Corydalis ophiocarpa* (Papaveraceae)

Ming Lei [1,2,3], Cui Li [1,2,3], Jing Wang [1,2,3], Mei Qin [1,2,3], Li-rong Huang [1,2,3], Xia-lian Ou [1,2,3], Liang Kang [1,2,3,4], Han Liu [1,2,3,*] and Zhan-jiang Zhang [1,2,3,5,*]

1 Guangxi Key Laboratory of Medicinal Resources Protection and Genetic Improvement, Guangxi Botanical Garden of Medicinal Plants, Nanning 530023, China; leiming@gxyyzwy.com (M.L.); licuicui941@163.com (C.L.); 18565756016@139.com (J.W.); qinmei20210630@163.com (M.Q.); huanglirong2066@126.com (L.H.); 18290045835@163.com (X.O.); 17736623451@163.com (K.L.)
2 National Center for Traditional Chinese Medicine (TCM) Inheritance and Innovation, Guangxi Botanical Garden of Medicinal Plants, Nanning 530023, China
3 Guangxi Engineering Research Center of TCM Resource Intelligent Creation, Guangxi Botanical Garden of Medicinal Plants, Nanning 530023, China
4 School of Pharmacy, Guangxi Medical University, Nanning 530021, China
5 Guangxi Key Laboratory for High-Quality Formation and Utilization of Dao-di Herbs, Guangxi Botanical Garden of Medicinal Plants, Nanning 530023, China

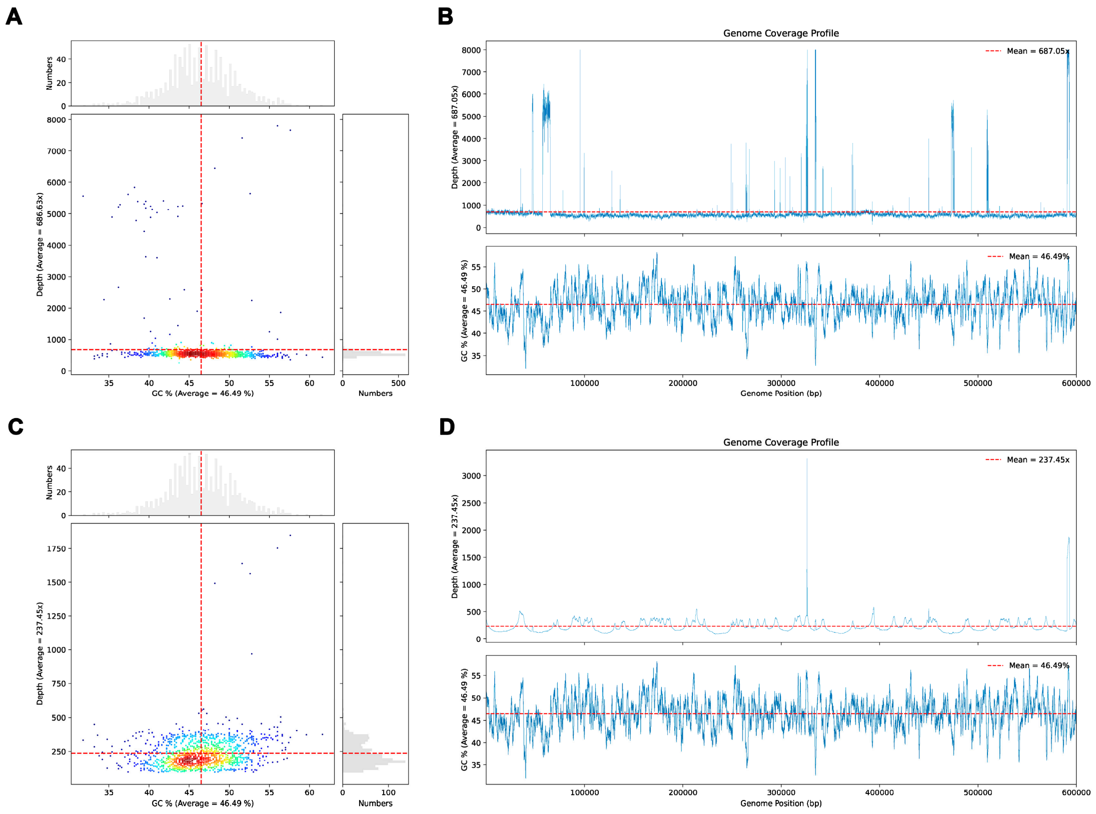

Supplementary Figure S1. The GC content, sequncing depth, and coverage depth of the *C. ophiocarpa* mitochondrial (mt) genome. (**A**) The density distribution of GC content versus sequencing depth of the Illumina reads across the *C. ophiocarpa* mt genome. Sliding windows of 500 bp without overlap were utilized to calculate the GC content (%) and sequencing depth (defined as the sum of sequencing depths across all positions within the window divided by the window size) for each window. Each point represents a single window, with point color determined by Gaussian kernel density estimation, while warmer colors indicate a higher concentration of windows with the corresponding GC-depth combination. The top histogram illustrates the GC content distribution of all windows across the mt genome, while the right histogram displays the depth distribution. Red dashed lines denote the mean GC content and mean sequencing depth of the mt genome, respectively. (**B**) The genome-wide coverage depth and GC content profile of the Illumina reads across the *C. ophiocarpa* mt genome. The upper panel presents the per-base sequencing depth across the mt genome, and the lower panel illustrates the GC content profile. GC content was calculated using a centered sliding window spanning 500 bp upstream and 500 bp downstream of each position (total window size = 1 kb), sampled at 1-bp intervals. Red dashed lines indicate the mean sequencing depth and mean GC content of the mt genome, respectively. (**C**) The density distribution of GC content versus sequencing depth of the Oxford Nanopore reads across the *C. ophiocarpa* mt genome. (**D**) The genome-wide coverage depth and GC content profile of the Oxford Nanopore reads across the *C. ophiocarpa* mt genome.

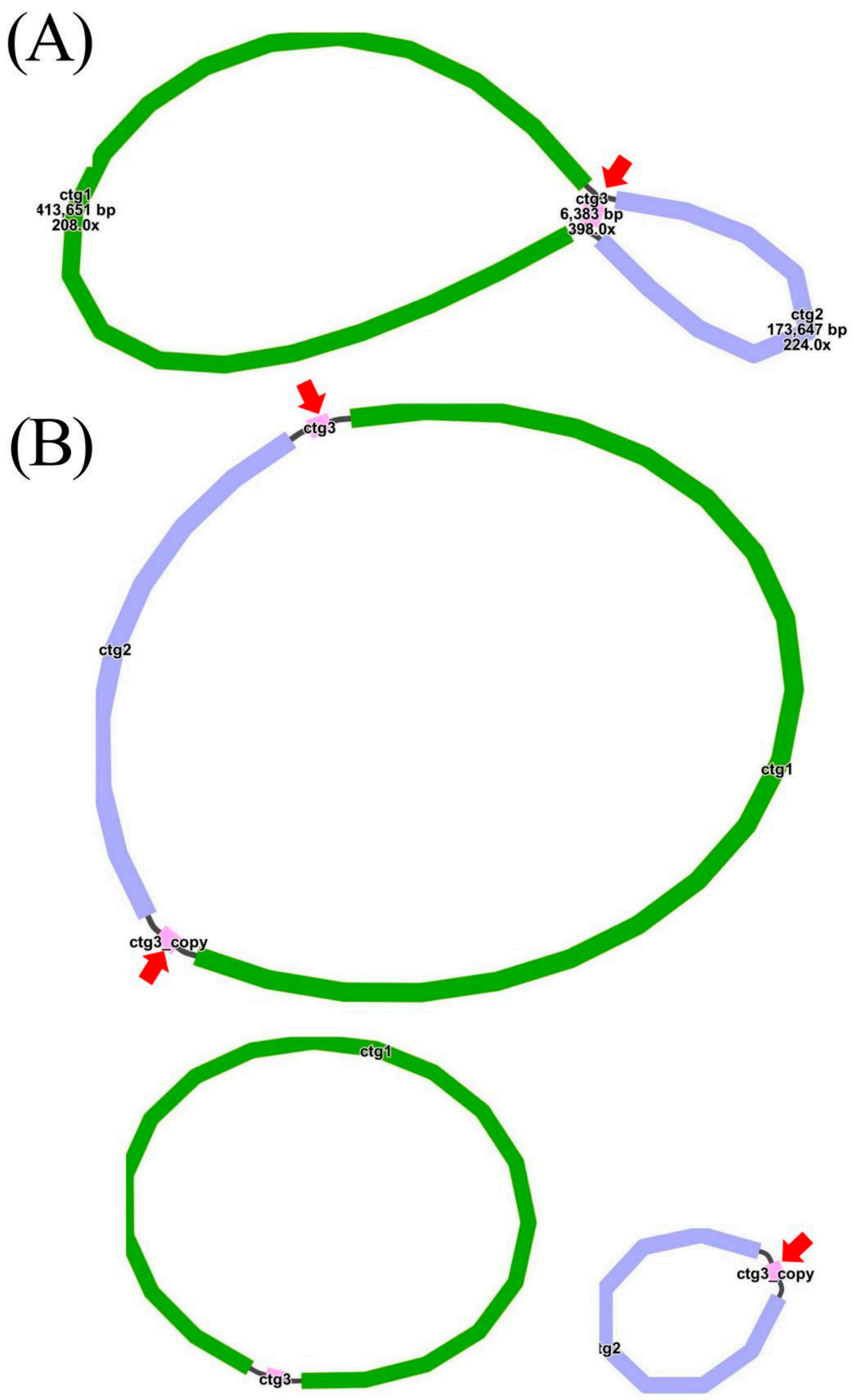

Supplementary Figure S2. Dynamic multipartite architecture of the *C. ophiocarpa* mitogenome. (A) The master circle of the *C. ophiocarpa* mitogenome. (B) Recombinogenic subgenomic conformations derived from the master circle via the 6383 bp repeat.

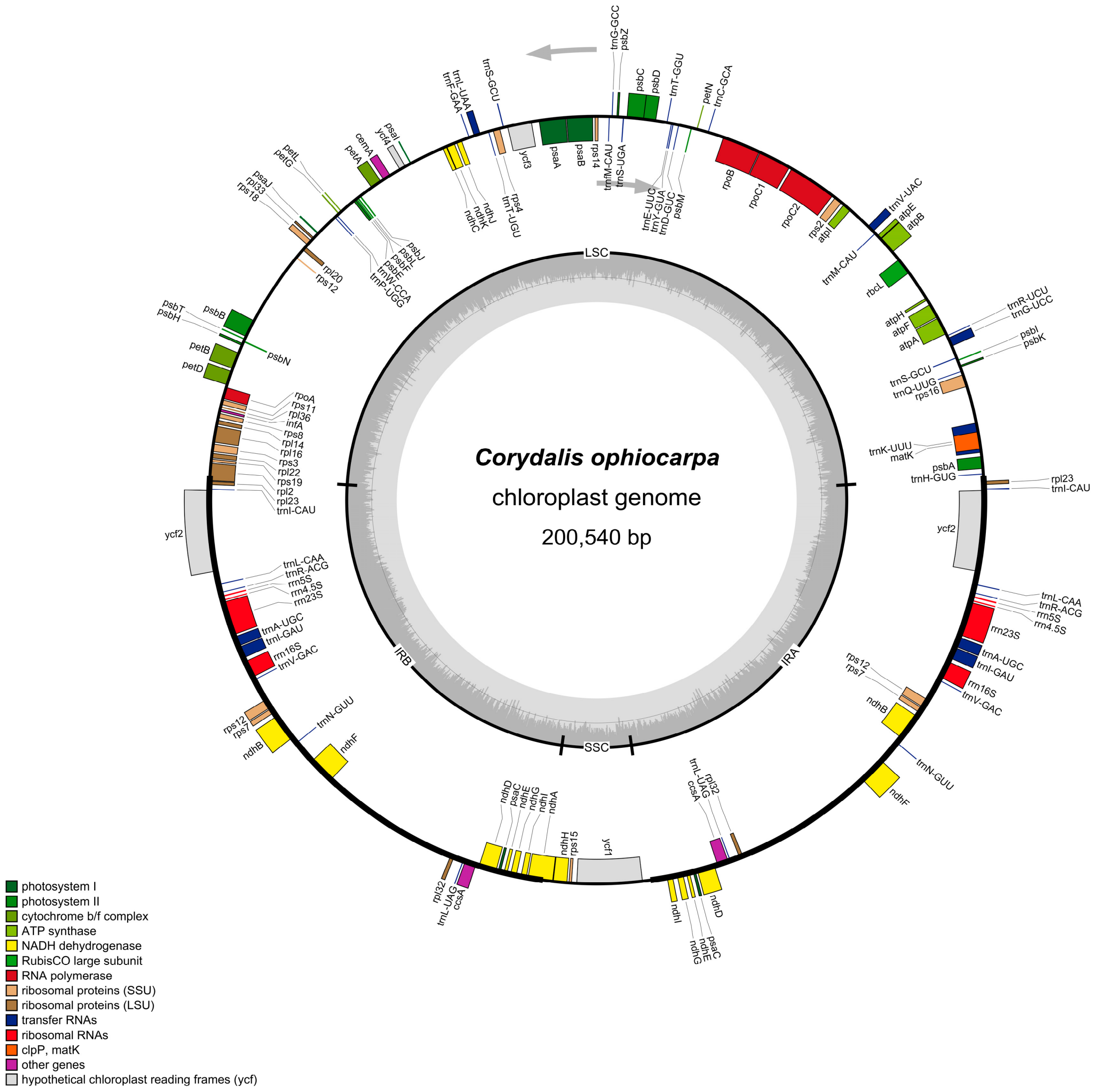

Supplementary Figure S3. A schematic representation of the *C. ophiocarpa* chloroplast genome. The inner circle displays the GC content distribution, with dark grey shading representing GC content and a light black line indicating the 50% GC threshold. The outer circle illustrates the circularized genomic sequence. The genes located on the interior and exterior of the circle are transcribed in a clockwise and counterclockwise direction, respectively.

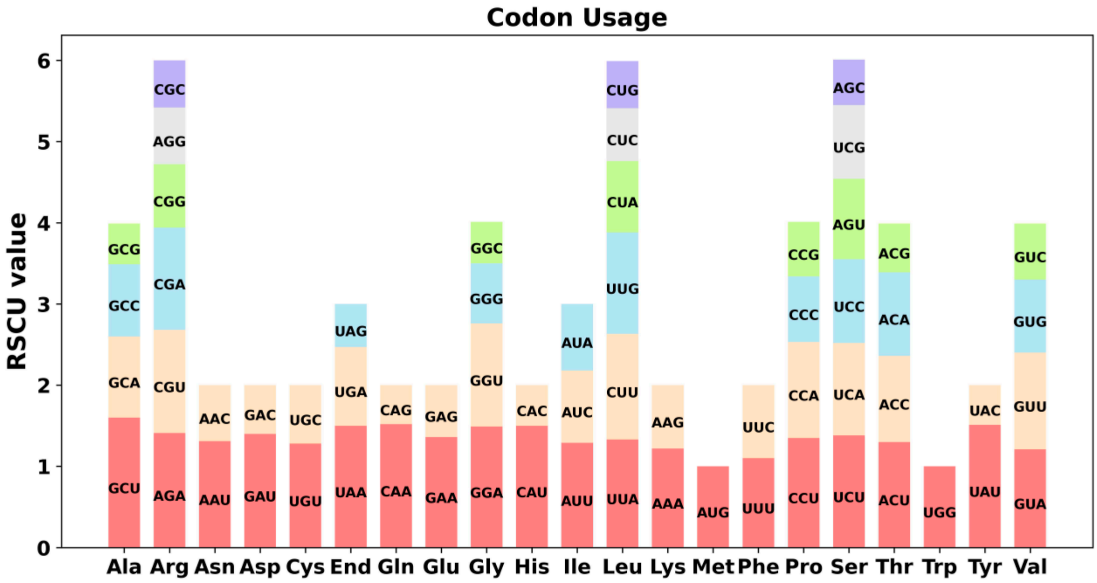

Supplementary Figure S4. Codon usage of 20 amino acids and stop codons across all mt protein-coding genes (PCGs) of *C. ophiocarpa*. The histogram employs different colors to represent the codons.

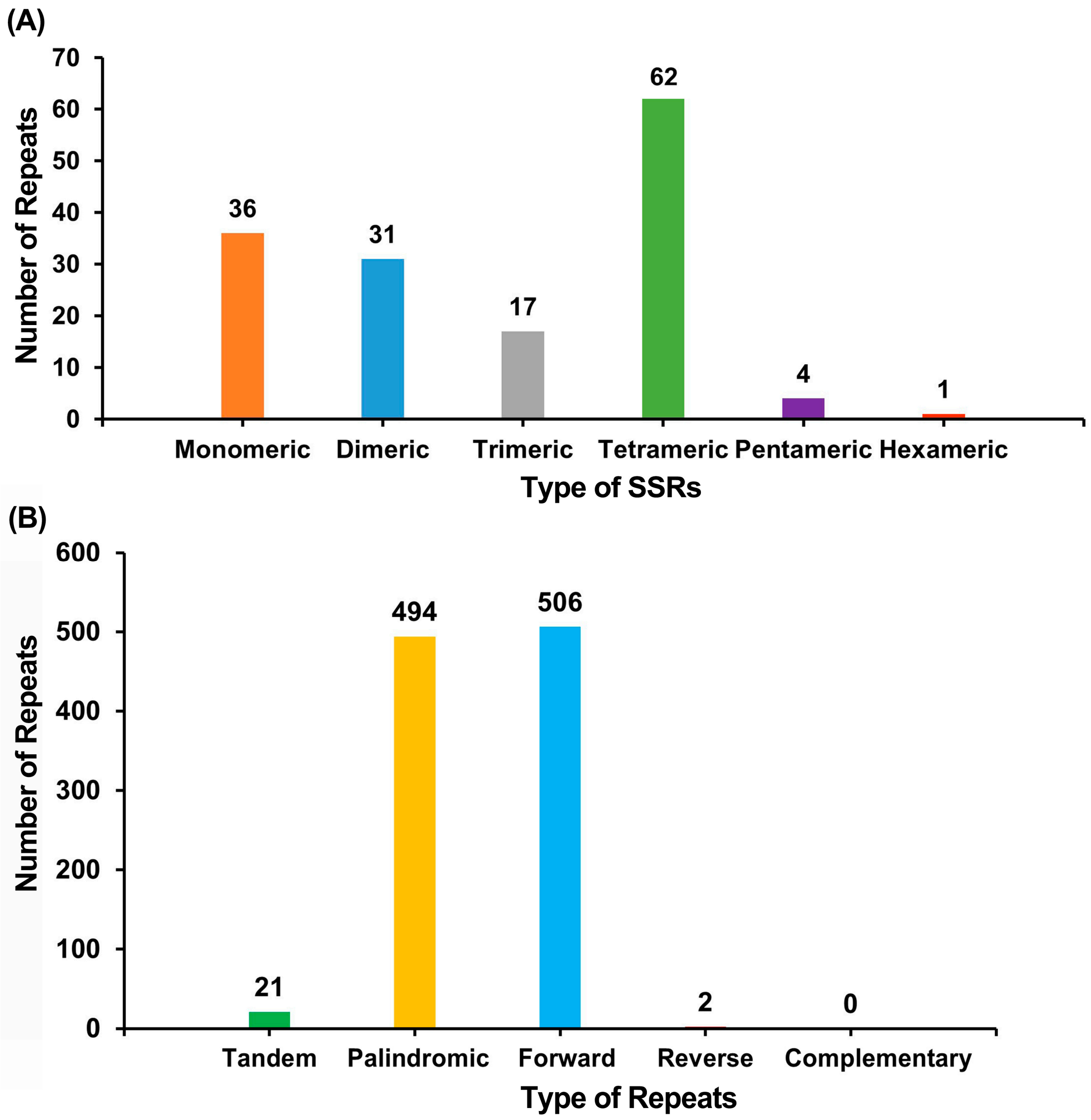

Supplementary Figure S5. Repeated sequences in the *C. ophiocarpa* mt genome. (**A**) Distribution of SSRs in the *C. ophiocarpa* mt genome. (**B**) Distribution of tandem and dispersed repeats in the *C. ophiocarpa* mt genome.

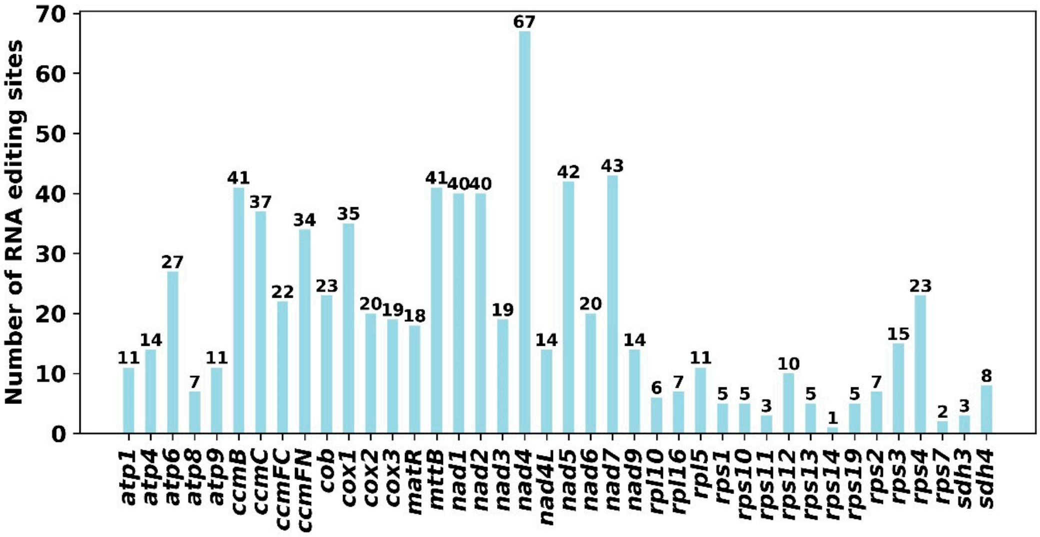

Supplementary Figure S6. The distribution of RNA-editing sites across the 40 PCGs within the mt genome of *C. ophiocarpa*. The numbers positioned above each group indicate the quantity of RNA-editing sites present in the corresponding genes.
